# Single-arm, first-in-human feasibility study results for an ultra-low-cost insulin pump

**DOI:** 10.1186/s12902-024-01652-y

**Published:** 2024-08-01

**Authors:** Matthew Payne, Francis Pooke, Tom M. Wilkinson, Lui Holder-Pearson, Bronté Chamberlain, Martin de Bock, J. Geoffrey Chase

**Affiliations:** 1 Department of Mechanical Engineering, Centre for Bioengineering, University of Canterbury, 20 Kirkwood Avenue, Christchurch, 8041 New Zealand; 2 Department of Electrical and Computer Engineering, University of Canterbury, 20 Kirkwood Avenue, Christchurch, 8041 New Zealand; 3 Department of Paediatrics, University of Otago, Christchurch, New Zealand

**Keywords:** Insulin pump, Open-source, Low-cost, Clinical trial

## Abstract

**Background:**

Use of Continuous Subcutaneous Insulin Infusion (CSII) has been shown to improve glycemic outcomes in Type 1 Diabetes (T1D), but high costs limit accessibility. To address this issue, an inter-operable, open-source Ultra-Low-Cost Insulin Pump (ULCIP) was developed and previously shown to demonstrate comparable delivery accuracy to commercial models in standardised laboratory tests. This study aims to evaluate the updated ULCIP in-vivo, assessing its viability as an affordable alternative for those who cannot afford commercially available devices.

**Methods:**

This first-in-human feasibility study recruited six participants with T1D. During a nine-hour inpatient stay, participants used the ULCIP under clinical supervision. Venous glucose, insulin, and β-Hydroxybutyrate were monitored to assess device performance.

**Results:**

Participants displayed expected blood glucose and blood insulin levels in response to programmed basal and bolus insulin dosing. One participant developed mild ketosis, which was treated and did not recur when a new pump reservoir was placed. All other participants maintained β-Hydroxybutyrate < 0.6 mmol/L throughout.

**Conclusion:**

The ULCIP safely delivered insulin therapy to users in a supervised inpatient environment. Future work should focus on correcting a pump hardware issue identified in this trial and extending device capabilities for use in closed loop control. Longer-term outpatient studies are warranted.

**Trial Registration:**

The trial was prospectively registered with the Australian New Zealand Clinical Trials Registry (ACTRN12623001288617) on the 11 December 2023.

## Background

Use of Continuous Subcutaneous Insulin Infusion (CSII), otherwise termed insulin pumps, has been shown to improve glycemic outcomes in Type 1 Diabetes (T1D) [1–4], therefore reducing the risk of associated complications and improving quality of life [5, 6]. However, a recent small scale study (*n* = 61) showed CSII use in young children (mean age of 4.9) may not give a statistically significant improvement in H_b_A_1c_ [7].

Automated insulin delivery, which requires an insulin pump is recognised as gold standard for the management of T1D [8, 9]. However, the high associated cost of CSII [10] means this technology is underutilised, particularly in countries without reimbursement from either insurance or government. This can lead to health inequity, where those with low socio-economic status do not access gold standard management, and are over represented in poor health outcomes [11, 12]. There is therefore an important need to develop insulin pumps which can be manufactured and supplied at substantially lower prices.

Bench-side dose accuracy results for an Ultra Low Cost Insulin Pump (ULCIP) were presented in the Journal of Diabetes Science Technology in December 2022 [13], with an in depth presentation of the open-source hardware published in HardwareX [14]. The design is deliberately inter-operable, targeted towards eventual use in automated insulin delivery. These results show it is possible to create an insulin pump from low-cost components (BOM < US$100) offering comparably accurate insulin delivery to commercial models when tested in a laboratory environment with standardised tests [15].

**Fig. 1:**
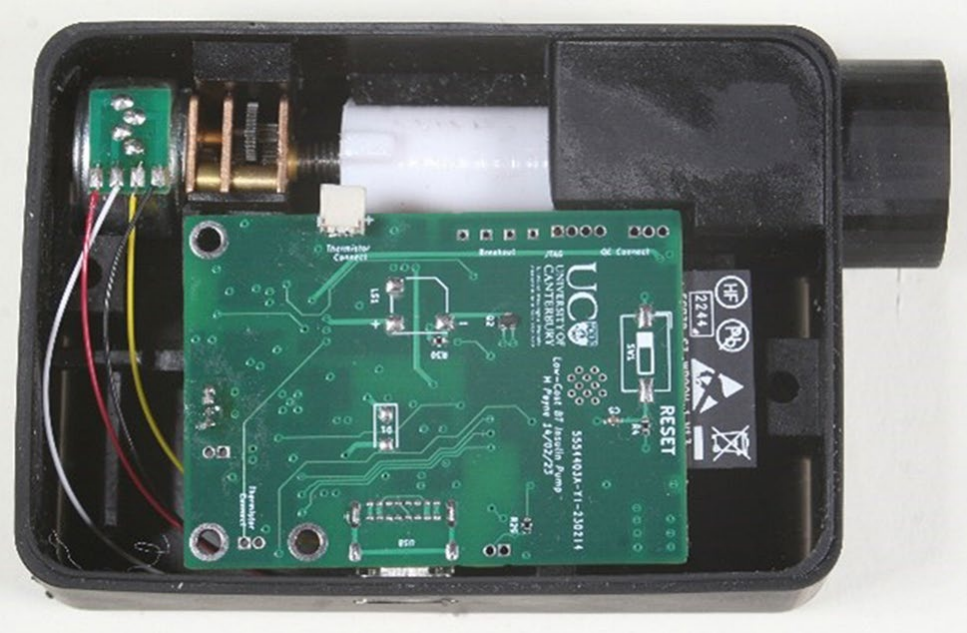
A photo of the ULCIP used in the clinical trial

Since the original publication and design, an updated version of the ULCIP has been designed, which utilises Bluetooth connectivity for pump control, as well making other improvements to the design presented previously [13, 14]. The total cost of parts for this updated device is $80USD. An image of the updated device is shown in Fig. 1 below:

The aim of this study is to document the performance of this updated design by trialling the ULCIP in-vivo.

## Methods

This single-arm first-in-human feasibility study was conducted in Ōtautahi/Christchurch, New Zealand, in December 2023. Following screening and informed consent, six participants with T1D were enrolled.

Inclusion criteria were: age > 18 years, a diagnosis of T1D as per the American Diabetes Association (ADA) classification [16], current use of insulin pump therapy with a basal rate within the range able to be programmed into the ULCIP, and glycated hemoglobin (H_b_A_1c_) < 97mmol/mol (11.0%) based on the mean of all available results within the last six months.

Exclusion criteria were: a positive pregnancy test or currently breastfeeding, any episodes of severe hypoglycaemia or diabetic ketoacidosis within the past six months, allergy or intolerance to Humalog^®^ and NovoRapid^®^ insulin, self-reported alcohol or drug dependence, or the presence of any other comorbid medical or psychological factors which in the opinion of investigators would render a person unsuitable for the study.

Participants attended an initial study visit (day 1) where they underwent a baseline assessment including collection of basic demographic and anthropometric data, current pump settings, and history of diabetes control. Participants commenced use of a Dexcom G7 Continuous Glucose Monitor (CGM), which was used unblinded. CGM recordings were used to provide descriptive data regarding baseline glycemic control and to assist clinical management while using the ULCIP.

The inpatient phase of the trial commenced on day 6 (± 2 days). Participants attended a clinical trials unit, where they used the ULCIP under clinical supervision for nine hours. Alongside CGM monitoring, a venous blood sample was drawn hourly to check Blood Glucose (BG) and β-Hydroxybutyrate (β-OH-B) using a combined BG and Ketones meter (Caresens Dual, i-Sens, Seoul, South Korea), and to measure Blood Insulin (BI) values. Insulin was measured using the Siemens Attelica assay, which has been reported as able to detect insulin aspart and lispro with > 75% cross-reactivity [17].

ULCIP settings were selected by an on-site diabetes physician following review of existing pump settings and CGM data. The ULCIP was then initiated, using Medtronic MiniMed™ Sure T™ infusion sets and cannulae, and MiniMed™ 1.8 ml cartridges (Medtronic, Northridge, California) Each participant used either insulin NovoRapid^®^ (aspart) or Humalog^®^ (lispro). The ULCIP reservoir for all participants was intentionally under filled with 20 units insulin at the start.

During the nine-hour inpatient stay, participants were offered two meals, both containing at least 40 g of carbohydrates. Meals were timed so the corresponding insulin bolus was given 30 min prior to a scheduled blood test, allowing for serum insulin measurements 30 and 90 min after the bolus. Additional insulin boluses were given as required, at discretion of the supervising diabetes physician. After 9 h of use, a final blood sample was obtained, and participants were assisted in resuming their usual insulin pump regimen.

This is a first in human feasibility study, therefore formal comparative statistical analyses were not planned. All data collected in this study was documented using summary tables, plots and participant data listings.

## Results

All 6 participants completed the 9-hour inpatient in-vivo testing of the ULCIP. Participant demographics are shown in Table 1.

**Table 1:** Patient demographics

Participant Number	Age	Height (cm)	Weight (kg)	BMI	Mean TDD (IU)	H_b_A_1c_ (mmol/mol)
P1	63	171.6	85.6	29.1	56.8	40
P2	30	162.8	70.0	26.4	37.2	52
P3	31	180.9	104.4	31.9	74.3	69
P4	20	176.9	105.7	33.8	104.5	75
P5	24	167.5	76.6	27.3	41.8	47
P6	22	175.8	80.1	29.1	49.3	40

TIR for each participant is given in Table 2 for the range between 3.9 mmol/L to 8 mmol/L.

**Table 2:** TIR 3.9-8mmol/L comparison for each participant during in-vivo testing

Participant Number	Intra-trial TIR (%)
1	42.73
2	51.92
3	0.83
4	27.52
5	99.07
6	75.23

Participant 3 had a very low time spent in time in range. However, their glucose levels did not escalate during the study, and there was no significant rise in ketones. This reflects entering the study on a high glucose, and baseline pump settings that were not optimised for correction of moderate hyperglycaemia.

**Fig. 2:**
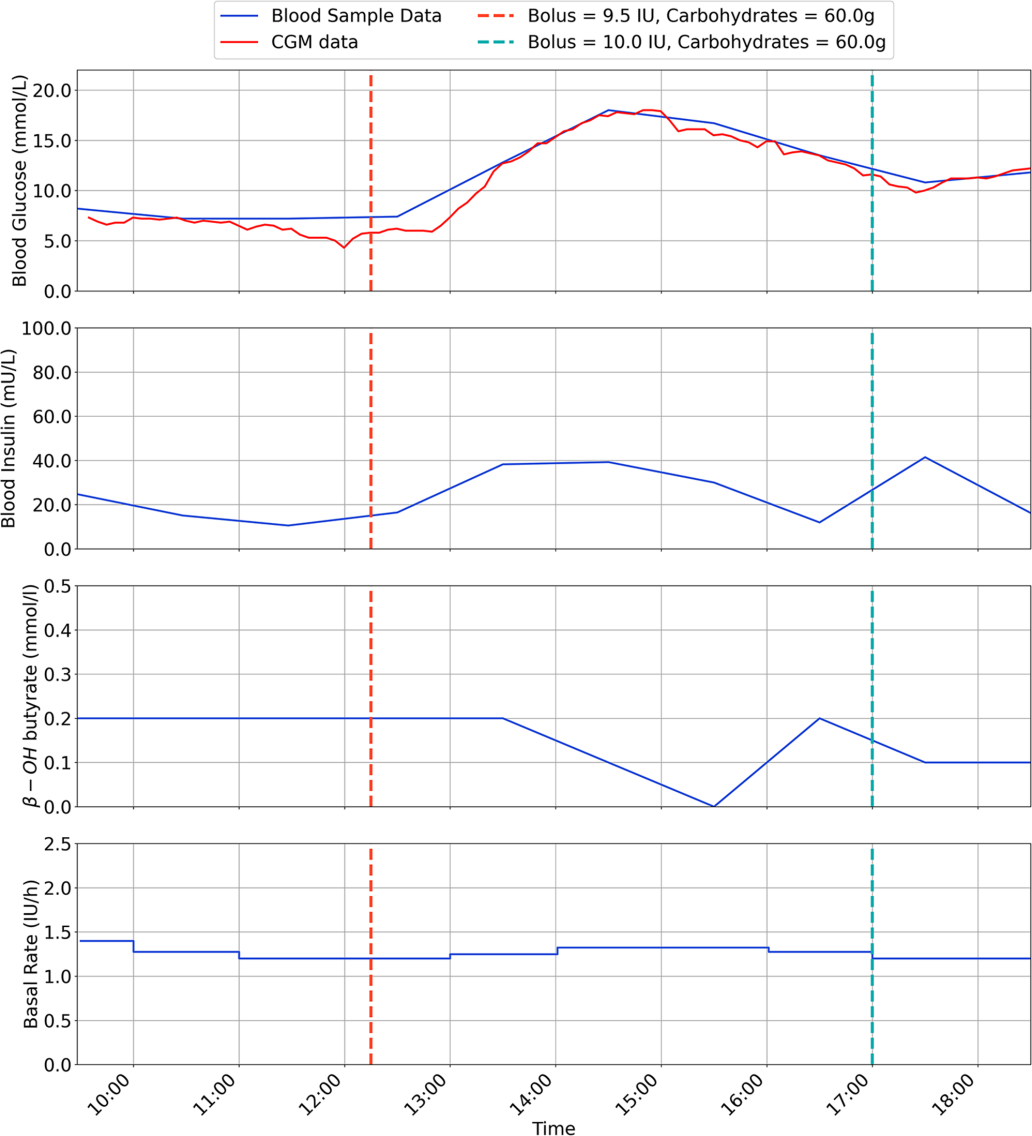
Inpatient trial data for participant 1

**Fig. 3:**
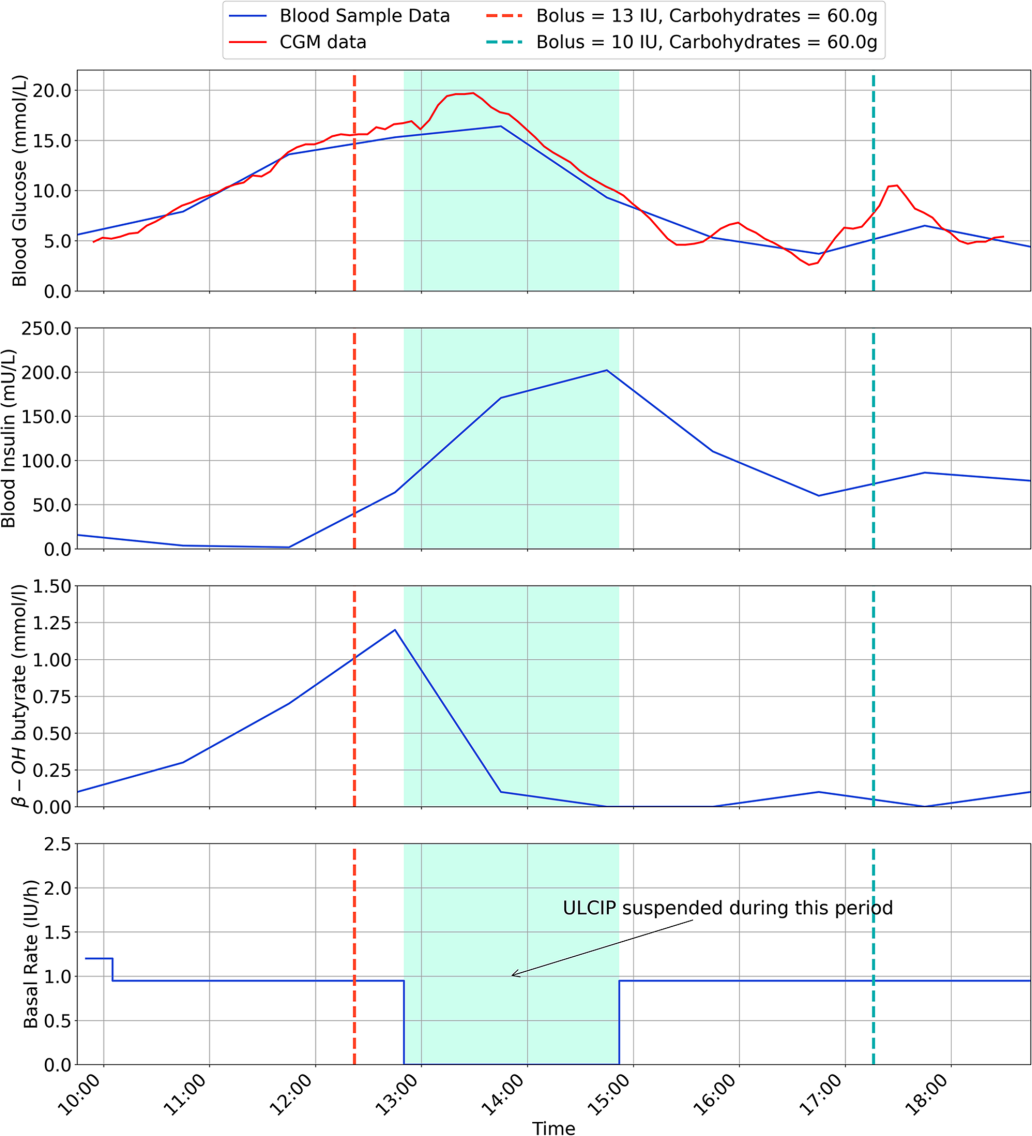
Inpatient trial data for Participant 2, with ULCIP suspended during green shaded time period

**Fig. 4:**
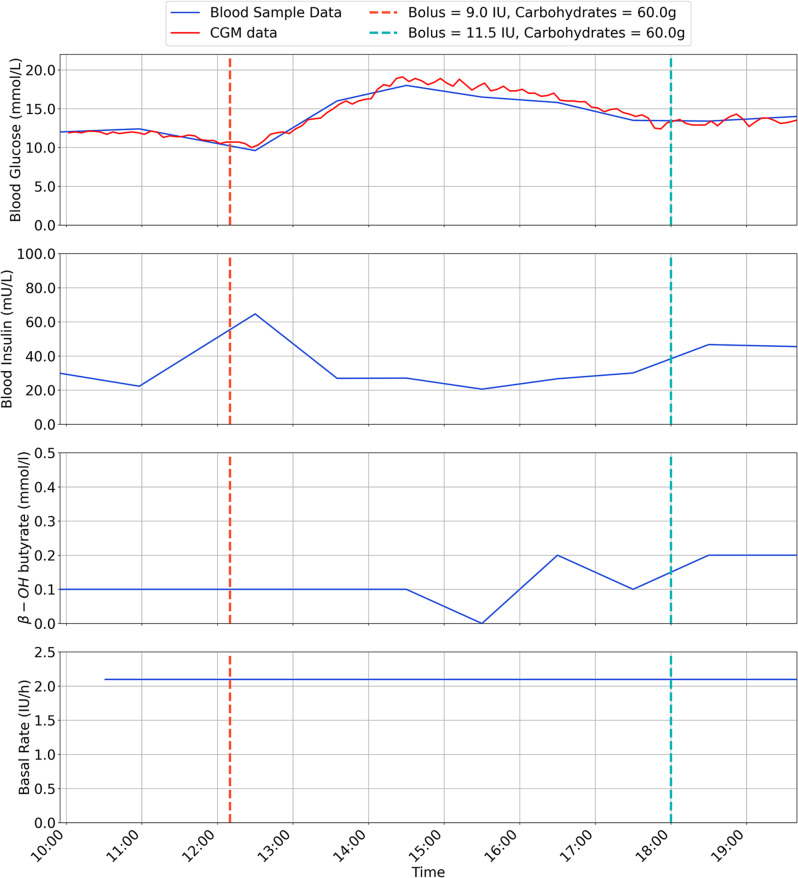
Inpatient trial data for participant 3

**Fig. 5:**
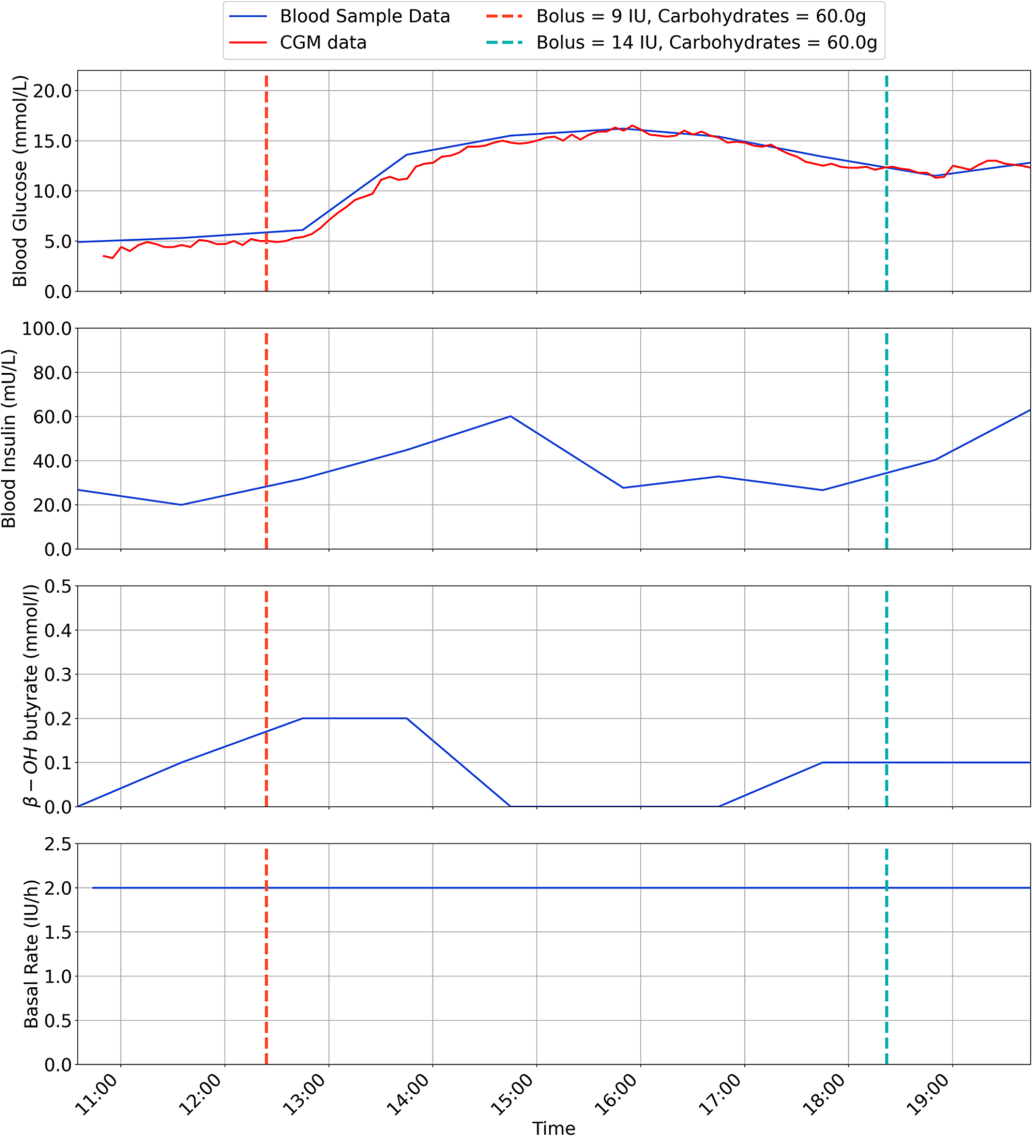
Inpatient trial data for participant 4

**Fig. 6:**
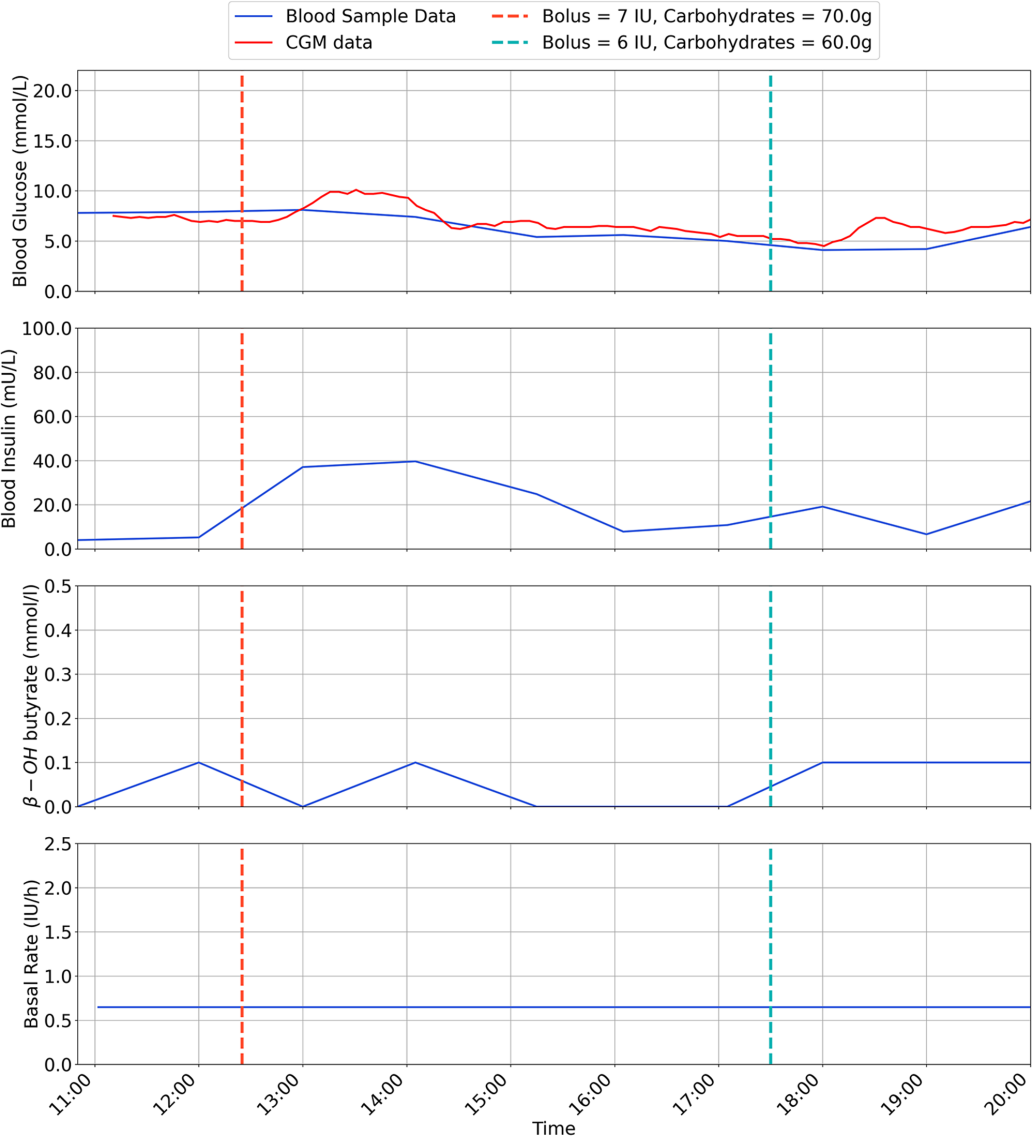
Inpatient trial data for participant 5

**Fig. 7:**
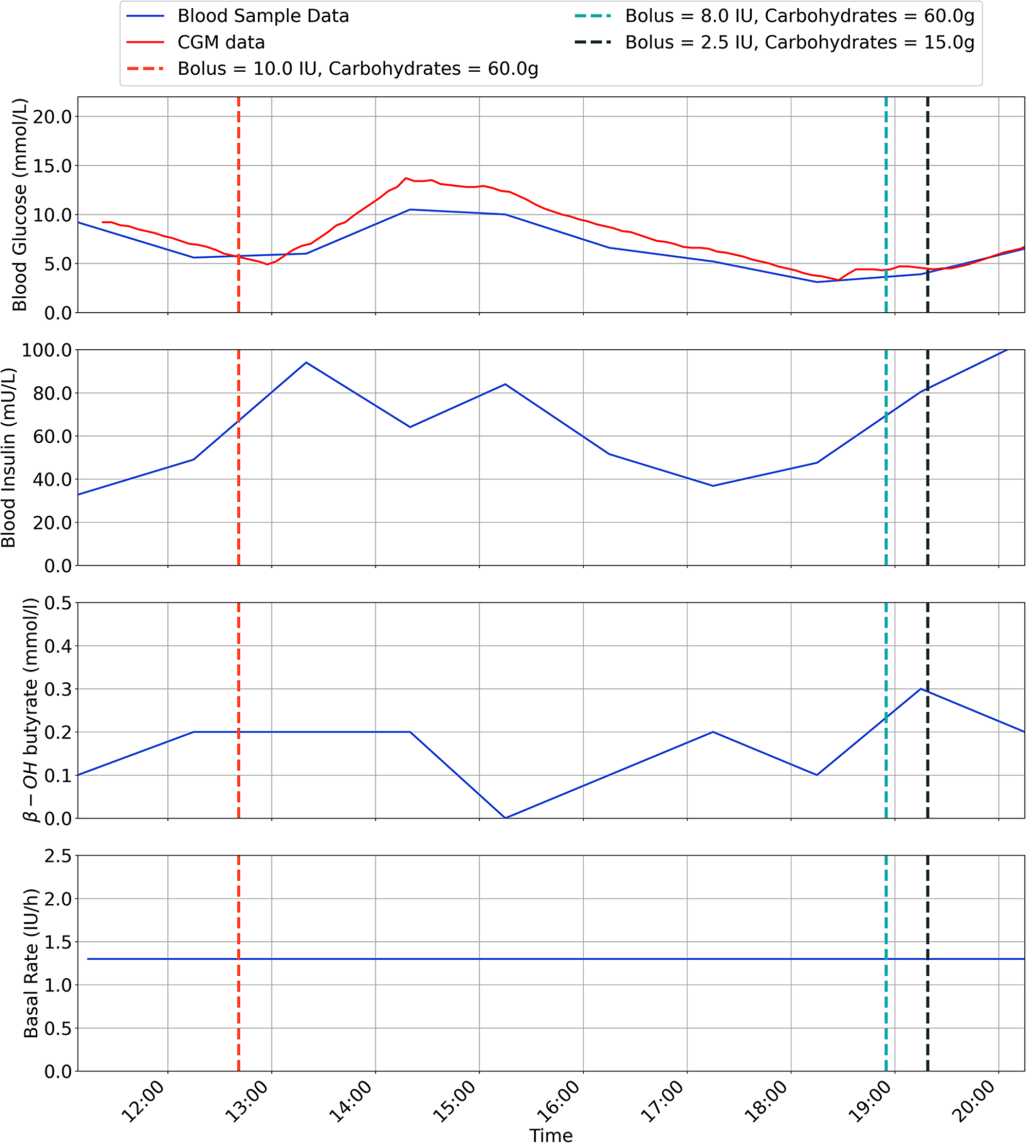
Inpatient trial data for participant 6

The results are for each participant are shown in Figs. 2, 3, 4, 5, 6, 7, with separate plots given for blood glucose (mmol/L), blood insulin (mU/L), β-OH-B (mmol/L) and programmed basal rate (IU/h). Dashed vertical lines mark the approximate time carbohydrates (g) and a bolus (IU) were given to each participant along with bolus size.

With respect to safety, one participant (P2) developed ketosis after 3 h use of the ULCIP (β-OH-B 1.2 mmol/L), following which the ULCIP was suspended and the participant resumed their usual insulin pump, with subsequent resolution of ketosis.

It was speculated that P2 had developed ketosis due to pump’s plunger operating towards the limit of its range when the reservoir was intentionally under-filled (as a pre-emptive safety measure to avoid severe hypoglycaemia from unintentional insulin release), resulting in inconsistent insulin delivery. Following this event, all new reservoirs placed in the ULCIP for all participants were more completely filled with approximately 90 units of insulin. Following resolution of ketosis, P2 was able to resume ULCIP use for 4 h (with a more completely filled reservoir), with β-OH-B remaining < 0.6 mmol/L during this time. No further episodes of ketosis were observed after this change to ULCIP use in any participants.

## Discussion

Drawing definitive conclusions from this study about the effectiveness of the device based on such a limited sample size and a short time frame presents challenges. Given these constraints, a key metric for success in this trial is the ULCIP’s ability to deliver the insulin dosage as prescribed by the diabetes clinician and indicated by observed changes in patients’ blood values aligning with the expected outcomes of the insulin delivery. In particular, if the device is effective in delivering insulin, then it would be expected to have similar efficacy in diabetes management to other pumps which have been shown to improve control and outcomes [1–4].

Firstly, the environment in which the ULCIP was tested is very different to how insulin pumps are used in a non-clinical setting. During the trial, participants were kept under a close watch with blood values taken hourly to ensure their device was functioning correctly.

Participants also had diabetes clinicians available to assist with correct bolusing and any changes in pump settings. Participants also did not exercise or undertake any physical activity, which may have meant changes in bolus sizes or basal rates. This highly controlled environment, while useful for safety and eliminating unwanted variables when testing a novel device, has few similarities to real world use, and this is important to remember to place these results in context.

The ULCIP has not undergone extensive failure testing. It is possible with long term usage some components of the device may be prone to failure. The ULCIP would need to undergo failure testing as required for certified medical devices to mitigate this risk. Additionally, because the ULCIP design has been made open source, the quality of the final product may vary depending on the production processes used (3D printing, PCB assembly). It is then difficult to generalise the results from this study, as any replicated designs may not have been built with similar tolerancing as those used in the clinical trial. However, an open pump allows design and safety improvements, as well as improvements to its connectivity and interface for use, all of which would enable low-cost closed loop systems and algorithms which have already demonstrated efficacy [18, 19].

User experiences outside of the clinical trial environment may be different, as the cohort participating in this study are likely to be more open to using new technologies, since they volunteered for a trial of a novel device. This group is then likely to be non-representative of the general population of those with diabetes, who may be more conservative in their approach to new and less ‘proven’ technologies and so less likely to be interested, However, as noted, an open pump increases equity of access due to lower cost and greater availability, so this issue can be addressed over time.

In terms of implications on research, this small positive result for the ULCIP2.1 warrants a larger trial. This would be the logical next step in validating the safety and efficacy of the ULCIP2.1, and make the results more generalisable.

Regarding implications for practice, these trial results are the first step towards the inclusion of a low-cost insulin pump being available for endocrinologists to offer their patients. Even if qualification criteria for a government or insurance funded pump remained stringent, the low cost of these devices would mean an increase in equity, due to their greater affordability. Consequently, there would be greater equity amongst those with diabetes, leading to better health outcomes, regardless of the capacity of an individual to pay for treatment.

We have identified an important limitation of the ULCIP design, where underfilling the insulin reservoir results in poor engagement of the piston. By not underfilling the reservoir this issue was resolved. Nevertheless, this raised important questions regarding the design and safe use of this pump when the insulin volume in the reservoir is very low. Possible modifications could be made to the lead screw or plunger length, or alerts to the user when the insulin volume is low, alerting them to the need to refill the reservoir (much like commercial pumps do now).

Subsequent research and development is planned, including resolving the hardware issue mentioned above. As we look to further improve this technology in the future, a key goal remains the incorporation of a fully closed-loop system by incorporating connectivity with a CGM and an open-source automated insulin delivery algorithm, such as Android APS.

**Fig. 8:**
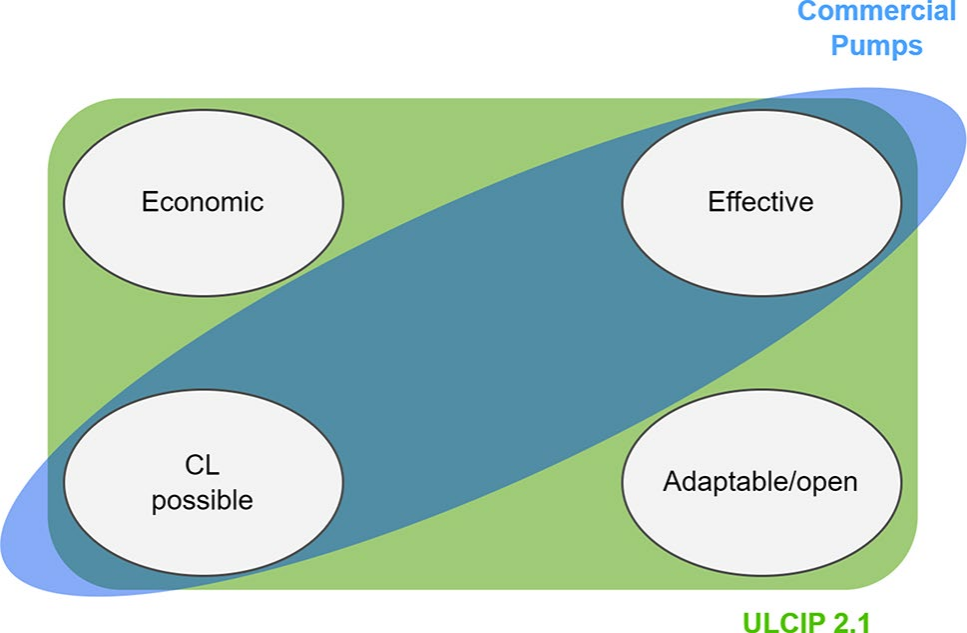
Pictorial summary of implications and results

## Conclusions

Very low-cost hardware to create an insulin pump has potential to provide more affordable and equitable diabetes care. This study is an important step to realising this goal, however more in-vivo testing is needed before outpatient studies can be safely conducted. The implications of the main reulst of the paper are summarised pictorially in Fig. 8.

## Data Availability

All data generated or analysed during this study are included in this published article.

## Abbreviations

CSII: Continuous Subcutaneous Insulin Infusion
T1D: Type 1 Diabetes
TIR: Time in Range
ULCIP: Ultra-Low-Cost Insulin Pump
CGM: Continuous Glucose Monitor
ADA: American Diabetes Association
TDD: Total Daily Dose
BG: Blood Glucose
BI: Blood Insulin
β-OH-B: β-Hydroxybutyrate, (Px) Participant x
